# Insulin Requirements for Patients With COVID-19 Presenting With Diabetic Ketoacidosis

**DOI:** 10.7759/cureus.33258

**Published:** 2023-01-02

**Authors:** Ahmed Nagy, Kristine Sobolewski, Jessica Bente

**Affiliations:** 1 Pharmacy, Deborah Heart and Lung Center, Browns Mills, USA; 2 Pharmacy, Cooperman Barnabas Medical Center, Livingston, USA

**Keywords:** insulin infusion, diabetes, diabetic ketoacidosis, covid-19, insulin

## Abstract

Introduction

Recent literature has shown that patients with COVID-19 and diabetic ketoacidosis may require more aggressive treatment than those with diabetic ketoacidosis alone. The primary objective of this study was to assess if intravenous regular human insulin infusion requirements in patients with diabetic ketoacidosis differed between patients with or without COVID-19.

Methods

This retrospective cohort study evaluated patients with diabetic ketoacidosis who received intravenous regular human insulin infusion during the COVID-19 pandemic. The primary outcome was the amount of intravenous regular human insulin infusion requirements needed during the diabetic ketoacidosis episode.

Results

Of the 77 patients that met inclusion criteria, 35 were positive for COVID-19 and 42 were negative. The primary outcome of total intravenous regular human insulin infusion requirements needed during the diabetic ketoacidosis episode was not statistically significant and resulted in 1.79±0.61 units/kg/day in the COVID-19 positive group and 1.81±0.6 units/kg/day in the negative group (p=1). Secondary outcomes that were statistically significant between groups were the amount of fluids received in the first 24 hours, potassium supplementation, phosphate supplementation, acute kidney injury, and hypokalemia.

Conclusion

There was no difference in intravenous regular human insulin infusion requirements in the setting of diabetic ketoacidosis (DKA) between COVID-19 positive and negative patients.

## Introduction

Severe acute respiratory syndrome coronavirus 2 (SARS-CoV-2) was first identified in November 2019 and is the causative pathogen implicated in the development of COVID-19. COVID-19 causes respiratory illness and creates a systemic inflammatory storm. Comorbidities, such as diabetes mellitus (DM), increase the risk of severe illness from COVID-19, which may lead to a higher risk of complications, such as diabetic ketoacidosis (DKA) [1]. DKA is a type of hyperglycemic crisis that is characterized by the triad of uncontrolled hyperglycemia, metabolic acidosis, and increased total body ketone concentration. Precipitating factors include discontinuation of insulin or oral antihyperglycemic therapy, viral or bacterial infection, pancreatitis, myocardial infarction, and other diseases that accompany a significant bodily stress response. Treatment includes fluid resuscitation, insulin therapy, potassium and phosphate supplementation, and sodium bicarbonate in severely acidotic patients [2].

COVID-19 has been shown to precipitate DKA in patients with DM, cause hyperglycemia in patients with an unknown history of DM, and potentially induce new-onset DM [3,4]. A single-center, retrospective chart review showed that many patients that had COVID-19 and DKA did not have poorly controlled DM before admission, implying that COVID-19 could lead to hyperglycemic crises regardless of DM diagnosis [5]. Several medical institutions globally have also observed that COVID-19 contributes to tremendous insulin requirements, but to what extent is unclear [6].

Due to the mostly qualitative and anecdotal nature of the current literature available examining DKA in COVID-19-positive patients, this study was designed to quantify intravenous (IV) regular human insulin (RHI) infusion requirements between DKA patients that were COVID-19 positive versus those that were COVID-19 negative.

## Materials and methods

This IRB-approved retrospective cohort study was conducted at a large, community teaching hospital. The IRB waived the need for informed consent due to the retrospective nature of the study. Patients evaluated were at least 18 years of age, diagnosed with DKA, and received IV RHI infusion during the initial surge of the COVID-19 pandemic (February 1, 2020, to June 30, 2020). None of the patients were vaccinated as a vaccine was not available during this time frame. Patients were identified using drug charge codes for IV RHI infusion through a local data mining platform. DKA diagnoses were confirmed by ensuring that each patient had a plasma glucose concentration of ≥250 mg/dL, had a pH of ≤7.3 and/or a serum bicarbonate concentration of ≤18 mEq/L, and the presence of ketones in the urine and/or serum. Patients found to have DKA were further delineated into two cohorts, COVID-19 positive and COVID-19 negative. Patients were deemed positive based on COVID-19 lab results or provider documentation of high suspicion of disease. Providers used clinical diagnostics, labs (e.g., ferritin, D-dimer), or chest X-rays in the event of limited testing availability and delayed turnaround times.

At this large, community teaching hospital, providers treated patients with DKA using a hospital protocol. DKA severity was categorized as mild (pH ≥7.21, serum bicarbonate ≥15 mEq/L), moderate (pH 7.01-7.2, serum bicarbonate 9-14 mEq/L), and severe (pH ≤7, serum bicarbonate ≤8 mEq/L). Vital signs were initially checked every 30 minutes with continuous pulse oximetry. Complete blood count on presentation, comprehensive metabolic panels on presentation and at 4 hours, and venous blood gas profile on presentation and at 4 hours were performed for all patients. Blood glucose monitoring, via point-of-care testing, occurred hourly initially and then every 2 hours once patients were more stable. Bolus hydration, if administered, was with 0.9% sodium chloride and doses ranged from 500 mL to 2000 mL or providers could choose a 30 mL/kg option. Continuous IV hydration was divided into two groups: patients with a corrected serum sodium concentration <140 mEq/L (0.9% sodium chloride) or patients with a corrected serum sodium concentration ≥140 mEq/L (0.45% sodium chloride). This fluid was administered initially at a rate of 125 mL/hr and providers had the option to include 20-40 mEq of potassium per liter bag to maintain a serum potassium concentration of 3.5-5 mEq/L. IV RHI infusion was administered at a rate of 0.1 units/kg/hour and had a concentration of 100 units per 100 mL of 0.9% sodium chloride. Fluids containing dextrose 5% in water were started once blood glucose concentrations were <300 mg/dL. The IV RHI infusion did not stop until the following criteria were met: anion gap ≤16 (calculation includes potassium) for at least 4 hours, blood glucose concentration ≤200 mg/dL for at least 4 hours, and the patient has received an appropriate subcutaneous (subQ) dose of long-acting insulin at least 2 hours before discontinuation of IV RHI infusion.

The primary outcome was the amount of IV RHI infusion requirements administered during the DKA episode. Secondary outcomes were divided into three main categories: insulin treatment characteristics, DKA management, and safety. Insulin treatment characteristic outcomes included total IV RHI infusion requirements used for the duration of DKA treatment, IV RHI infusion requirements in the first 24 hours, total IV RHI infusion and subQ insulin used during DKA treatment (subQ doses were included if administered during IV RHI infusion), time to IV RHI infusion initiation, time on IV RHI infusion, and first subQ basal insulin dose. DKA management outcomes included the amount of fluids used in the first 24 hours, average daily fluid usage, potassium and phosphate supplementation during DKA episode, time to glucose control (defined as a blood glucose concentration ≤200 mg/dL), time to document anion gap closure (defined as anion gap ≤16), anion gap at the end of IV RHI infusion, final anion gap documented, and intensive care unit (ICU) and hospital length of stay. Safety outcomes included the presence of acute kidney injury or AKI (based on provider diagnosis), hypoglycemia (defined as a blood glucose concentration <70 mg/dL), hypokalemia (defined as a serum potassium concentration <3.5 mEq/L), and time to mortality (days from admission date).

All eligible patients during the specified time period were included for statistical analysis. Baseline characteristics were analyzed using descriptive statistics. Continuous data were analyzed with an unpaired student t-test, if parametric, or a Mann-Whitney U test, if nonparametric. Ordinal data were analyzed with a Chi-square test. Where data were missing, we report the number of available observations, and we make no assumptions about the missing data. A five percent level of significance was used for the data collected and specific outcomes were considered to be statistically significant if the p-value was less than 0.05.

## Results

**Figure 1:**
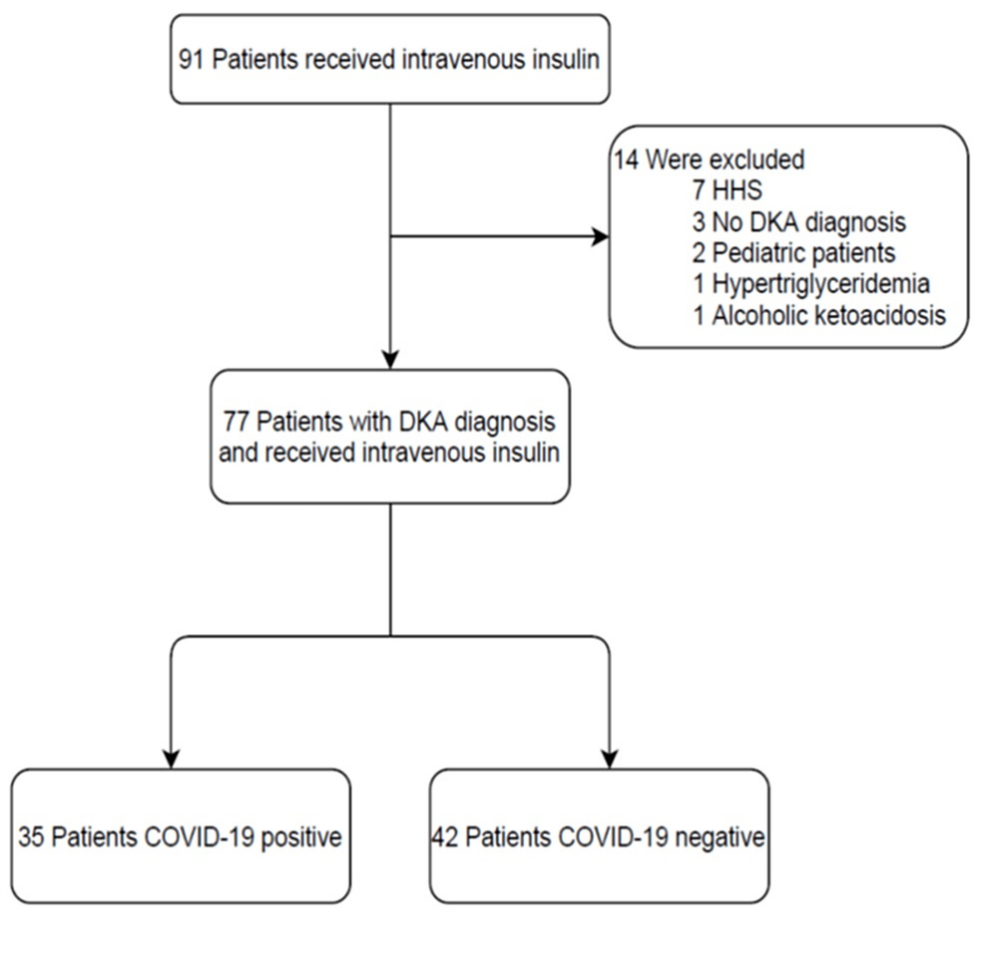
Evaluation and Study Groups HHS: Hyperosmolar hyperglycemic syndrome, DKA: Diabetic ketoacidosis, COVID-19: Coronavirus disease 2019.

Between February 1, 2020, and June 30, 2020, a total of 91 patients received IV RHI infusion, of whom 77 were evaluated for clinical outcomes (35 were COVID-19 positive and 42 were COVID-19 negative) (Figure 1). Baseline characteristics were largely similar between the two groups (Table 1). The baseline characteristics that differed between COVID-19 positive and negative patients, respectively, were SOFA score (6±4 versus 3±2), total prednisone-equivalent steroid dose (125.93±290.39 mg versus 8.69±33.37 mg), and new-onset DM (two patients or 6% versus 10 patients or 24%). The reasons for admission for each patient varied, however, that data was not collected. Initial vital signs and laboratory values, upon presentation or earliest value, were also generally similar between the two groups (Table 2). Respiratory rate was higher in the COVID-19 positive group than the COVID-19 negative group, 31 breaths/min versus 24 breaths/min, respectively. Blood urea nitrogen (BUN) was higher in the COVID-19 positive group than the COVID-19 negative group, 51.3±33 mg/dL versus 36.7±24.9 mg/dL, respectively.

**Table 1:** Baseline Characteristics COVID-19: Coronavirus disease 2019, BMI: Body mass index, DM: Diabetes mellitus, T1DM: Type 1 diabetes mellitus, T2DM: Type 2 diabetes mellitus, COPD: Chronic obstructive pulmonary disease, SOFA: Sequential organ failure assessment.

Characteristic	COVID-19 Positive (n=35)	COVID-19 Negative (n=42)
Age – years (mean ± SD)	58±15	52±20
Male sex – no. (%)	21 (60%)	28 (66%)
Actual weight – kg (mean ± SD)	89±20	81±29
BMI – kg/m­^2 ^(mean ± SD)	30.7±6.6	28.2±8.5
Race – no. (%)		
Black	28 (80%)	25 (60%)
White	4 (11%)	8 (19%)
Asian	0 (0%)	7 (17%)
Other	3 (9%)	2 (4%)
Type of DM – no. (%)		
T2DM	31 (88%)	25 (60%)
T1DM	1 (3%)	7 (17%)
None	3 (9%)	10 (24%)
Past medical history – no. (%)		
Asthma	2 (6%)	2 (5%)
COPD	1 (3%)	3 (7%)
Cardiovascular disease	24 (69%)	26 (62%)
Liver disease	0 (0%)	2 (5%)
Kidney disease	6 (17%)	10 (24%)
Dialysis-dependent	0 (0%)	4 (10%)
Prior to admission antidiabetic medications – no. (%)		
Insulin	17 (35%)	18 (43%)
Oral agents	20 (57%)	13 (31%)
SOFA score (mean ± SD)	6±4	3±2
Total prednisone-equivalent steroid dose – mg (mean ± SD)	125.93±290.39	8.69±33.37
New-onset DM – no. (%)	2 (6%)	10 (24%)

**Table 2:** Initial Vital Signs and Laboratory Values COVID-19: Coronavirus disease 2019, BUN: Blood urea nitrogen, HbA1c: Hemoglobin A1c.

Initial Vital Sign/Laboratory Value	COVID-19 Positive (n=35)	COVID-19 Negative (n=42)
Mean arterial pressure – mm Hg (mean ± SD)	70±15	74±13
Heart rate – beats/min (mean ± SD)	122±20	115±26
Respiratory rate – breaths/min (mean ± SD)	31±8	24±5
Oxygenation saturation – % (mean ± SD)	87±13	95±2
Temperature – °F (mean ± SD)	100.1±1.8	98.8±0.6
Potassium – mmol/L (mean ± SD)	4.7±0.8	4.5±1
Blood glucose – mg/dL (mean ± SD)	597±265	641±297
BUN – mg/dL (mean ± SD)	51.3±33	36.7±24.9
Creatinine clearance – mL/min (mean ± SD)	60±43	69±55
HbA1c – % (mean ± SD)	12.7±2.8 (n=23)	11.5±2.7 (n=35)
Urine ketones noted – no. (%) (mean ± SD)	27 (77%)	33 (79%)
pH (mean ± SD)	7.257±0.133 (n=32)	7.203±0.115
Anion gap (mean ± SD)	27±7	30±8
Beta-hydroxybutyrate – mg/dL (mean ± SD)	55.4±44.3 (n=26)	56.6±37.9 (n=33)
C-reactive protein – mg/dL (mean ± SD)	16.3±11.1 (n=26)	5.8±11.2 (n=8)
D-dimer – ng/mL (mean ± SD)	3561±3928 (n=24)	1170±1891 (n=6)
Ferritin – ng/mL (mean ± SD)	1686±1078 (n=21)	523.5±298.8 (n=8)
Lactic acid – mmol/L (mean ± SD)	2.62±0.91 (n=25)	3.53±2.8 (n=27)
Procalcitonin – ng/mL (mean ± SD)	4.05±9.38 (n=23)	2.68±5.94 (n=13)

The primary outcome of the amount of IV RHI infusion requirements needed during the DKA episode was not statistically significant resulting in 1.79±0.61 units/kg/day in the COVID-19 positive group and 1.81±0.6 units/kg/day in the COVID-19 negative group (p=1) (Table 3). For the insulin treatment characteristic secondary outcomes, there were no statistically significant results between the two groups (Table 3). The total IV RHI infusion requirements used was 112±196 units versus 82±50 units (p=0.99) in the COVID-19 positive and COVID-19 negative groups, respectively. Time on IV RHI infusion was 24.7±69.1 hours versus 14.2±8.9 hours (p=0.65) and the first basal insulin dose was 0.2±0.2 units/kg versus 0.3±0.1 units/kg (p=0.06) between the COVID-19 positive and COVID-19 negative groups, respectively.

**Table 3:** Study Outcomes COVID-19: Coronavirus disease 2019, IV: Intravenous, RHI: Regular human insulin, subQ: Subcutaneous, DKA: Diabetic ketoacidosis, ICU: Intensive care unit, AKI: Acute kidney injury.

Outcome	COVID-19 Positive (n=35)	COVID-19 Negative (n=42)	P-value
PRIMARY OUTCOME			
Total IV RHI infusion requirement – units/kg/day (mean ± SD)	1.79±0.61	1.81±0.6	1
SECONDARY OUTCOMES			
Insulin			
Total IV RHI infusion used – units (mean ± SD)	112±196	82±50	0.99
IV RHI infusion used in first 24 hr – units (mean ± SD)	73±46	75±47	0.87
Total IV RHI infusion and subQ – units/kg/day (mean ± SD)	2.14±0.8	2.23±0.87	0.65
Time to IV RHI infusion – hr (mean ± SD)	5.9±8.6	4.4±4.5	0.3
Time on IV RHI infusion – hr (mean ± SD)	24.7±69.1	14.2±8.9	0.65
First basal insulin dose – units/kg (mean ± SD)	0.2±0.2	0.3±0.1	0.06
DKA management			
Fluids used in first 24 hr – L (mean ± SD)	3.75±1.73	4.74±2.02	0.02
Average daily fluid usage – L/day (mean ± SD)	1.57±1.62	1.71±0.92	0.07
Potassium use during DKA episode – mEq (mean ± SD)	23.8±40.2	68.4±86.9	<0.01
Phosphate use during DKA episode – mmol (mean ± SD)	7.7±28.9	19.4±31.2	<0.01
Time to glucose control (≤200) – hr (mean ± SD)	10.1±10.1 (n=33)	9±13.6	0.37
Time to anion gap closure (≤16) – hr (mean ± SD)	20.6±30.4 (n=32)	11.8±17.4	0.18
Anion gap at end of IV RHI infusion (mean ± SD)	16±4	15±3	0.12
Final anion gap documented (mean ± SD)	15±4	14±4	0.69
ICU length of stay – days (mean ± SD)	2.8±4.5	1.1±1.2	0.23
Hospital length of stay – days (mean ± SD)	10.9±14	5.2±3.5	0.06
Safety			
Presence of AKI – no. (%)	25 (71%)	20 (48%)	0.03
Presence of hypoglycemia – no. (%)	2 (6%)	8 (19%)	0.1
Presence of hypokalemia – no. (%)	4 (11%)	14 (33%)	0.02
Time to mortality – days (mean ± SD)	6.1±6.1 (n=16)	7.2±6.3 (n=3)	0.65

The DKA management secondary outcomes that showed a significant difference between COVID-19 positive and COVID-19 negative patients were the amount of fluids used in the first 24 hours (3.75±1.73 L versus 4.74±2.02 L; p=0.02), potassium use during the DKA episode (23.8±40.2 mEq versus 68.4±86.9 mEq; p <0.01), and phosphate use during the DKA episode (7.7±28.9 mmol versus 19.4±31.2 mmol; p <0.01) (Table 3). Time to anion gap closure was 20.6±30.4 hours (n=32) versus 11.8±17.4 hours (p=0.18), anion gap at the end of the IV RHI infusion was 16±4 versus 15±3 (p=0.12), and final anion gap documented was 15±4 versus 14±4 (p=0.69) when comparing the COVID-19 positive to the COVID-19 negative patients. The length of stay in the ICU was 2.8±4.5 days versus 1.1±1.2 days (p=0.23) and the length of stay in the hospital was 10.9±14 days versus 5.2±3.5 days (p=0.06) between the COVID-19 positive and COVID-19 negative groups, respectively.

The safety secondary outcomes that showed a significant difference between COVID-19 positive and COVID-19 negative patients were the presence of AKI (25 patients or 71% versus 20 patients or 48%; p=0.03) and the presence of hypokalemia (four patients or 11% versus 14 patients or 33%; p=0.02). Hypoglycemia occurred in two (6%) patients versus eight (19%) patients (p=0.1) and the time to mortality was 6.1±6.1 days (n=16) versus 7.2±6.3 days (n=3) (p=0.65) when comparing the COVID-19 positive to the COVID-19 negative patients, respectively.

## Discussion

This retrospective investigation identified that IV RHI infusion requirements did not differ between COVID-19 positive and COVID-19 negative patients that had DKA. COVID-19 and its impact on patients with DM is a topic of growing interest as there are theories about the injury to the beta cells of the pancreas, exaggerated cytokine response, activation of the renin-angiotensin system, and changes in health behaviors during the COVID-19 pandemic [7]. It has been theorized that SARS-CoV-2 binds to ACE2 receptors expressed in the pancreatic islets, which may lead to islet destruction and acute diabetes [8-10]. In a patient with type 2 DM, this could potentially lead to a state of insulinopenia and precipitate a DKA episode. A retrospective cohort study identified that COVID-19 can cause ketosis via fat breakdown and can lead to DKA in patients with DM [11]. This could vary patients’ responses to insulin as this effect may differ among patients depending on their baseline glucose control [12]. A systematic review identified that 77% of COVID-19 patients with DKA had T2DM, which corresponds to the findings in our study [13]. The variety of insulin requirements that have been recorded in the literature in patients with COVID-19 includes a case series that showed patients using up to 100 units/day, clinicians observing patients requiring 4 units/kg/day, and a case report that reported a patient requiring an infusion rate of 50 units/hr [9,10,13]. Another retrospective case series that evaluated 35 patients had shown that 12 patients (35%) required an increase in the fixed IV RHI infusion for DKA [14]. Compared to this literature, our study differed as we used a comparator group, COVID-19 negative patients, to quantify any differences that could be seen between groups.

In contrast, our study showed there was no significant difference in total IV RHI infusion requirements between COVID-19-positive and COVID-19-negative patients presenting with DKA. Interestingly, this was the case even though the COVID-19-positive group required higher total prednisone-equivalent steroid doses to control the “cytokine storm”, which would usually require greater insulin requirements to control the hyperglycemia caused by steroids [10,13]. This suggests that insulin management may not need to be more aggressive when managing a patient with both DKA and COVID-19, but rather that these patients are likely to require IV RHI infusion for a prolonged time. This is further evidenced by the fact that no other secondary outcomes related to insulin treatment characteristics were significantly different. This aligns with a retrospective cohort study that showed that insulin requirements did not differ between COVID-19 patients and non-COVID viral pneumonitis patients, which argues against the theory that SARS-CoV-2 attacks the pancreas [15].

Although not statistically significant, both total IV RHI infusion requirements and time on IV RHI infusion had a wider range of variability in the COVID-19 positive group compared to the COVID-19 negative group, which may be explained by a couple of reasons. In this community teaching hospital, IV RHI infusions are usually only administered in critical care settings but started to be used on different units, as the usual critical care units were fully occupied at the peak of the pandemic. This variability may be due to limited resources and an insufficient number of healthcare professionals to handle this surge of critically ill patients, the different providers’ familiarity with IV RHI infusions, and the acute nature of the COVID-19 pandemic. This challenge forced institutions to become more innovative and flexible with the way they treated DKA. During the early stages of the pandemic, many severely ill COVID-19 patients required several titratable parenteral medications, such as vasopressors and inotropes, so a shortage of infusion pumps occurred. As a method to deal with the possible large IV RHI infusion requirements, shortage of infusion pumps, and to reduce exposure to COVID-19 to healthcare professionals, healthcare staff was monitoring blood glucose concentrations less frequently once a patient was on a stable infusion rate (i.e., from every 1-2 hours to every 4-6 hours) [10]. Less frequent monitoring may have potentially led to larger IV RHI infusion requirements and time on IV RHI infusion, which could have caused the medical team to not realize that a DKA episode had resolved.

Although not statistically significant, the time to anion gap closure was longer in the COVID-19 positive group than in the COVID-19 negative group. In combination with the variability of some of the insulin treatment characteristic secondary outcomes, it further shows how the management of DKA in COVID-19 patients can vary. Although not evaluated, this may be due to the different levels of COVID-19 severity that these patients presented with. Anion gap closure could be harder to achieve since BUN was higher in the COVID-19-positive group. The presence of DKA and uremia, two potential causes of anion-gap metabolic acidosis, could make anion-gap closure harder to achieve. The presence of uremia can be explained by the higher incidence of AKI that was evident in the COVID-19-positive group. The length of stay in the ICU and hospital was longer in the COVID-19-positive group, which can be explained by intubation, sedation, and hemodynamic instability more so than the complicated management of a DKA episode.

This study was limited by a couple of factors. This was a retrospective, single-center cohort study with a relatively small sample size. In addition, AKI was defined based on diagnoses made in the patient charts rather than objective measures. Also, the number of patients evaluated for time to glucose control and documented anion gap closure was decreased due to early all-cause mortality and/or incomplete chart documentation. Lastly, the COVID-19-positive patients were not categorized into the severity of their disease (e.g., mild, moderate, or severe). As this study was completed prior to the availability of a vaccine, which can decrease COVID-19 severity, it is not possible to conclude if vaccination status would play a role in affecting the primary outcome.

## Conclusions

This retrospective cohort study did not identify a difference in IV RHI infusion requirements between patients with or without COVID-19 in the setting of DKA. Notably, longer durations of IV RHI infusion were noted in the COVID-19-positive group, which may warrant further investigation. Since this study does not align with most of the literature that shows greater insulin requirements in COVID-19-positive patients, future multi-center studies should be implemented to determine if there is a correlation between COVID-19 severity and greater IV RHI infusion requirements to manage a DKA episode.
